# Modulation of Aromatase by Phytoestrogens

**DOI:** 10.1155/2015/594656

**Published:** 2015-12-21

**Authors:** Edwin D. Lephart

**Affiliations:** Department of Physiology and Developmental Biology and The Neuroscience Center, Brigham Young University, Provo, UT 84602, USA

## Abstract

The aromatase enzyme catalyzes the conversion of androgens to estrogens in many human tissues. Estrogens are known to stimulate cellular proliferation associated with certain cancers and protect against adverse symptoms during the peri- and postmenopausal intervals. Phytoestrogens are a group of plant derived naturally occurring compounds that have chemical structures similar to estrogen. Since phytoestrogens are known to be constituents of animal/human food sources, these compounds have received increased research attention. Phytoestrogens may contribute to decreased cancer risk by the inhibition of aromatase enzyme activity and CYP19 gene expression in human tissues. This review covers (a) the aromatase enzyme (historical descriptions on function, activity, and gene characteristics), (b) phytoestrogens in their classifications and applications to human health, and (c) a chronological coverage of aromatase activity modulated by phytoestrogens from the early 1980s to 2015. In general, phytoestrogens act as aromatase inhibitors by (a) decreasing aromatase gene expression, (b) inhibiting the aromatase enzyme itself, or (c) in some cases acting at both levels of regulation. The findings presented herein are consistent with estrogen's impact on health and phytoestrogen's potential as anticancer treatments, but well-controlled, large-scale studies are warranted to determine the effectiveness of phytoestrogens on breast cancer and age-related diseases.

## 1. Introduction

The aromatase enzyme, a product of the CYP19A1 gene, catalyzes the conversion of androgens to estrogens in many human tissue sites [1–3]. The biosynthesis of estrogens plays a principal role in neoplastic formation, especially in women's health [2, 3]. For breast cancer, aromatase activity and its inhibition have become a focus of treatment historically. Also, current pharmaceutical agents classified as aromatase inhibitors characterize the importance of this enzyme in steroid biosynthesis due to the potent impact of the estrogen product [2].

Phytoestrogens are a group of plant derived naturally occurring compounds that have chemical structures similar to estrogen [4, 5]. Initially, the focus on phytoestrogens examined their ability to bind mammalian estrogen receptors [6, 7]. However, other studies investigated the influence of phytoestrogens on the human aromatase enzyme where inhibition of aromatase activity has been demonstrated [4, 5, 8]. Since phytoestrogens are known to be constituents of animal and human food sources, these compounds have received increased research attention because of their potential significance and applications in human cancers and other diseases [6, 9].

The biological properties of phytoestrogens are covered in this review, for the most part, in relationship to their ability to inhibit human aromatase enzyme activity and their applications to human cancers (especially breast cancer) and other aged-related diseases. Specifically, this review will outline (a) the aromatase enzyme with brief historical descriptions about its function, activity, and gene characteristics, (b) phytoestrogens, a group of plant derived naturally occurring compounds that have reference to their classification and applications to human health, and (c) a chronological coverage of aromatase activity modulated by phytoestrogens from the early 1980s to 2015. Each section will present a brief descriptive background for each topic, followed by how each area was derived along with human applications and/or analysis for the cited studies presented.

## 2. Aromatase

### 2.1. Historical Background

The discovery of estrogens dates back to around the 1920s [2, 10]. Other investigators in the 1930s isolated estrogens from endocrine tissues along with human and animal urine samples [2, 10]. This newly discovered compound was originally termed “theelin” and was later named estrone [10]. This provided the interest to study how estrogen synthesis was accomplished. As noted in other reports, Meyer et al. and Ryan in the 1950s along with others demonstrated the conversion of androgens to estrogens by endocrine tissues [11–13].

It was quickly established via animal experimentation and human diseases/disorders that estrogens induced growth or proliferation of reproductive tissues [10]. Later, Thompson and Siiteri in the 1970s showed the aromatization of androgens in human placental microsomes [14]. Also, during the 1970s, Grodin et al. reported the extraglandular aromatization of plasma androstenedione in men and women [15]. In one case, Hemsell et al. reported that the extraglandular aromatization of androgens resulted in the feminization of a prepubertal boy [16]. It was not until the 1980s that the human aromatase cytochrome P450 protein was extracted from placental microsomes [17]. Moreover, the aromatase crystal structure was not reported until 2009 [18].

Since these investigators realized very early in their studies the importance of aromatase enzyme activity on breast, ovarian, and endometrial cancers and the mechanism of gynecomastia, soon many studies examined (1) how via mechanistic biochemical (step-by-step) processes estrogen biosynthesis took place, (2) where aromatization occurred in the body, (3) much more importantly how to regulate or inhibit the aromatase enzyme, and, finally, (4) what was the molecular biology structure of aromatase and its tissue-specific expression and regulation.

### 2.2. The Aromatase Enzyme Reaction

The irreversible conversion of androgens to estrogens is catalyzed by the enzyme complex termed aromatase [1–3, 17, 19]. Notably, the C-19 sex steroids, androstenedione and testosterone, have unsaturated A-ring structures that can serve as substrates in the aromatase reaction [17, 19, 20]. Conversely, dihydrotestosterone (DHT), a potent androgen, has a saturated A-ring which cannot be aromatized [20]. The aromatase enzyme is comprised of a microsomal cytochrome P450 complex that is a product of the CYP19A1 gene that belongs to a superfamily of P450 genes [1–3, 17, 19]. In general, the aromatization of androgens to estrogens takes place in the endoplasmic reticulum and is classified as a mono- or mixed-function oxidase reaction [1–3, 17, 19, 20]. This involves three consecutive reactions: hydroxylation, oxidation, and demethylation [17]. A highly conserved heme domain of the protein forms a ligand acceptor site (with high substrate specificity) that is involved in the aromatase reaction, and both NAPDH and oxygen are required for this conversion to produce C-18 estrogens [1–3, 17, 19, 20].

### 2.3. Tissue-Specific Sites of Aromatization

In normal human tissues aromatase enzyme activity is found in gonadal tissues (testis-Leydig and Sertoli, ovary-granulosa cells, and corpus luteum), uterus, breast, prostate, epididymis, placenta, adrenal glands, liver, skin (fibroblasts and adipose), muscle (skeletal and smooth), vascular endothelium, bone, and brain (neurons and glial) references [2, 3, 17, 19, 20]. There are many articles and reviews that provide details on this topic which is beyond the scope of this review.

However, estrogen receptor alpha and/or estrogen receptor beta have been identified in all of the tissue sites listed above for aromatase, whereby the estrogen product can exert its potent sex steroid hormone actions [17]. In fact, 17*β*-estradiol is the most potent steroid hormone due to its powerful proliferative actions in many cellular sites and on the other hand it has protective effects in other organs and tissues, especially with aging such as osteoporosis, cardiovascular effects, and dementia in women [10, 21].

### 2.4. Aromatase Inhibitors

**Figure 1:**
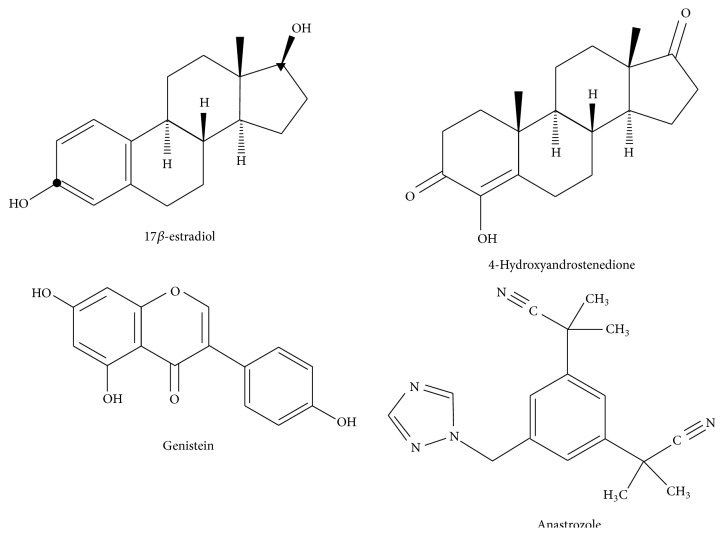
The chemical structure of 17*β*-estradiol, the most potent sex steroid hormone in the body, with carbon-3 indicated by ● and carbon-17 indicated by ▲, displays the functional groups associated with its biological activity; 4-hydroxyandrostenedione, one of the first aromatase inhibitors used in clinical trials; genistein, a phytoestrogen of the isoflavonoid class, and; anastrozole, a current pharmaceutical agent used as an aromatase inhibitor.

Hormone-dependent breast cancer and other endocrine disorders prompted investigators to develop potent and selective aromatase inhibitors. One approach was to target the aromatase enzyme using analogs of natural steroidal substrates in order to determine the structure/function relationship of the aromatase conversion process. In this regard, Marsh et al. examined many steroidal aromatase inhibitors that led to the discovery of 1,4,6-androstenedione and 4-hydroxyandrostenedione (4-OH-A) [22]. In fact, 4-OH-A became the first selective aromatase inhibitor for the treatment of breast cancer in the 1980s (see Figure 1) [2, 22, 23]. This avenue of research is closely related to how phytoestrogens have similar polyphenolic structures to that of 17*β*-estradiol and functional groups in a conformational pattern at carbon-3 and carbon-17 analogous to that of estrogens (see Figure 1) [4–6].

A second approach, based upon known endocrine interrelationships, was to block adrenal chemical messenger (steroid) function in order to treat breast cancer [2]. This latter approach led to the accidental finding that nonsteroidal P450 inhibitors were nonselective in the inhibition of aromatase but provided some therapeutic health benefit [2]. Ultimately, these studies lead to the utilization of aromatase inhibitors as highly effective treatments for breast cancer and reproductive disorders. Some of the current pharmaceutical aromatase inhibitors include anastrozole (see Figure 1), exemestane, and letrozole [21].

Also, aromatase activity has been shown to be regulated by phosphorylation, serine/threonine kinase inhibitors, protein kinase C inhibitors, tyrosine kinase inhibitors, and other steroidal hormones and agents that influence the second messenger pathway via cyclic AMP and other pathways [2, 17, 19].

In this review the main focus will be on how phytoestrogens, such as flavonoids, lignans, and other polyphenolic molecules, alter aromatase activity in relationship to cancer. However, other topics will also be considered where the implementation of plant estrogenic compounds may serve as treatment not only for cancer but also for other endocrine and age-related diseases.

### 2.5. Aromatase Gene Characteristics

This topic has been the subject of several reviews [2, 17, 19, 24] and will be presented here in brief. Aromatase is encoded by the CYP19A1 gene and there is no evidence for more than one isoform of aromatase existing in the human genome [17, 19, 24]. The human CYP19A1 gene is located on chromosome 15q21.1, which consists of nine coding exons (II to X) and a 5′-untranslated region forming an approximate length of 123 kb [17, 19]. To date, 11 tissue-specific promoters have been characterized that drive aromatase expression, where promoters 1.4, 1.7, 1.3, and II are associated with breast cancer [17]. Therefore, the expression of aromatase in humans is complex with tissue-specific promoters regulating the expression of the CYP19A1 gene. In general, in reference to breast cancer, aromatase expression is not regulated by estrogens [17]. However, there is evidence that steroids control aromatase expression in brain tissue sites along with human placental tissue [17].

## 3. Phytoestrogens

### 3.1. Classifications and Human Health Applications

Phytoestrogens represent a subclass of molecules under the polyphenol umbrella due to their chemical ring structures. Additionally, phytoestrogens denote a wide variety of compounds, which are divided into several classes, that is, hydroxybenzoic acids, hydroxycinnamic acids, anthocyanins, proanthocyanidins, flavonols, flavones, flavanols, flavanones, isoflavones, stilbenes, and lignans [25]. In higher plants, thousands of molecules have polyphenolic structures (e.g., several hydroxyl groups on aromatic rings) [26]. Polyphenols and/or phytoestrogens are secondary metabolites that are thought to be involved in protecting plants against ultraviolet radiation, aggression by pathogens, or stress-related responses such as drought [26, 27]. Several hundreds of these molecules are found in edible plants that include seeds, root, stem, leaf, and fruit portions of plants [26]. They are common micronutrients in the human diet and have been studied for their role in the prevention of cancer and cardiovascular diseases [25, 26]. Also, phytoestrogens have potential health benefits in age-related diseases and disorders due to their potent free radical scavenging properties and antioxidant activities [25, 26, 28].

**Figure 2:**
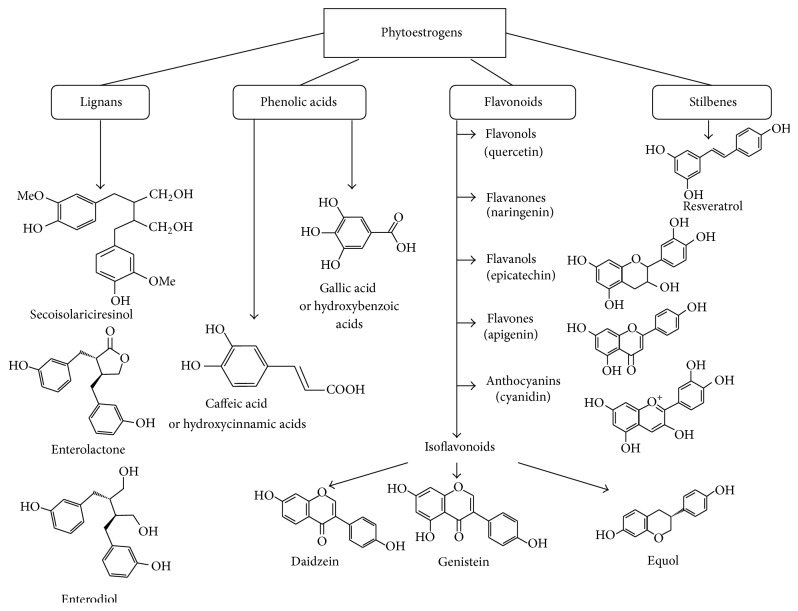
Classification and chemical structure of major classes of phytoestrogens.

Phytoestrogens are naturally occurring plant compounds, which are classified into several groups based upon their structure. The main classes include lignans, phenolic acids, flavonoids, and stilbenes [29].  Figure 2 displays the different major classes of dietary phytoestrogens with a representative name and most chemical structures for each group. Of all these groups, the stilbene resveratrol is the most high-profile polyphenol known to the general public. Following resveratrol, within the last decade or so, other polyphenols such as the flavonoids and especially isoflavonoids have a high profile in commercial food products along with the health benefits of the polyphenols found in tea (e.g., phenolic acids, flavonols, and catechins) [25, 26, 30, 31].

Novel insights of dietary polyphenols in the prevention of human disease and improving health covering such topics as cardiovascular, obesity, and inflammation/antioxidant effects, diabetes, and dermal protection have been reported [32–35].

### 3.2. Historical Background

Many phytoestrogens or polyphenolic compounds were discovered at about the same time as steroid hormones. For example, resveratrol, a polyphenolic stilbene, was first isolated by Takaoka in the 1940s [36]. Later Nonomura isolated resveratrol from Japanese knotweed (*Pediomelum cuspidatum*) in 1963 [37] with subsequent studies demonstrating that resveratrol was found in red grapes (wine) in 1992 by Siemann and Creasy [38]. Later, Bertelli et al., in 1995, reported that resveratrol had some cardioprotective effects [39]. However, there has been a dramatic increase in the number of journal articles about resveratrol since the discovery of its chemoprotective effects by John Pezzuto's laboratory in early 1997 [40]. This report showed the potential of resveratrol to prevent tumor initiation, promotion, and progression which may be used as a potential anticancer agent [41]. The publication history of resveratrol since this important discovery has shown a number of publications on the beneficial effects of resveratrol and calorie restriction, including the “French paradox,” where the abundant levels of resveratrol in red wines may be responsible for the surprisingly normal lifespan of the French in spite of their heavy consumption of fatty foods that can cause heart disease [41]. In addition to red grapes (wine), many other food products have been shown to contain natural resveratrol [41, 42].

Subsequent to the discovery by Pezzuto's laboratory on resveratrol's chemopreventive effects, more recent investigations during the last decade have focused on the mechanisms of how resveratrol acts especially in cell signaling paradigms covering cancer prevention, cardiovascular, and antiobesity along with its antioxidant and anti-inflammatory effects [41–43]. For example, resveratrol treatment not only covered cardiovascular protective actions but also alleviated diabetes-induced cardiovascular disorders via different endogenous signaling pathways including oxidative stress and glucose and insulin metabolism [44] as well as antidiabetic actions [45, 46]. In fact, a recent review by Park and Pezzuto in 2015 demonstrates the importance of resveratrol's pharmacological effects over the past five years (2009–2014) and covers a variety of research topics including cardiovascular, inflammation, carcinogenesis, the aging process, diabetes, neurological dysfunction, prostate, skin, and other disorders and diseases [47].

Other examples of the isoflavonoid class include daidzein, genistein, and equol. Equol was first isolated in 1932 and then identified approximately 50 years later in human urine as metabolite of the soy isoflavone daidzein [48]. These aglycones act like natural selective estrogen receptor modulators (SERMs) at various tissue sites throughout the body [48, 49]. Early on genistein received a great deal of research attention in the 1980s and throughout the 1990s due to its abundance and ready detection and quantification [49]. It was not until the equol hypothesis was proposed in the late 1990s that a shift in research focus was made toward equol [48]. Notably, the equol hypothesis suggests that the generation or consumption of equol above arbitrary threshold levels impacts health benefits for various disorders or diseases [48, 49]. Since this transition interval, there has been a dramatic increase in research publications on equol [48, 49].

Equol has many of the health benefits that are comparable to resveratrol, although equol, unlike its precursor daidzein (or genistein), is unique in having a chiral carbon at position C-3 of the pyran ring [48, 49]. It therefore can occur as two distinct isomers as* S*-equol or* R*-equol. Notably both isomers have unique antiandrogenic properties where they both specifically bind 5*α*-dihydrotestosterone (5*α*-DHT) [49, 50]. Equol is also a superior antioxidant, having greater antioxidant capacity than vitamin C or vitamin E [49], and it has many other benefits covering cosmetics, prostate, and breast cancer along with several other health applications [48–52].

Finally, it is not surprising that these phytoestrogens not only act as SERMs but also have the ability to alter the activity of the aromatase enzyme. Thus, the next sections deal with a brief historical coverage starting in the 1980s to 1999 followed by publications from 2000 up until 2014 of how various phytoestrogen compounds inhibit aromatase enzyme activity especially with regard to cancer. However, with the advancements in technology, the 2000 to 2014 interval displays a complex, evolving scientific investigative story.

### 3.3. 1980s–1999: Phytoestrogen Modulation of Aromatase Activity

In the 1980s, the pioneering work of Herman Adlercreutz along with Kenneth Setchell and other investigators demonstrated that dietary sources of nonsteroidal estrogen-like compounds had a profound impact on estrogen levels in women and the potential roles these molecules may play in hormone-dependent diseases, such as breast cancer [53–58]. This collective work sets up the stage for investigating how diet may influence disease such as cancer [6, 59].

One of the earliest reports on phytoestrogen compounds inhibiting aromatase activity was reported by Kellis Jr. and Vickery in 1984 who showed that several naturally occurring flavones altered estrogen biosynthesis [60] (see Table 1). Later in the late 1980s and into the 1990s, along with the development of steroid analogs as aromatase inhibitors [2, 73, 74], other investigators examined naturally occurring lignan and flavonoid compounds that inhibited aromatase activity in human preadipocytes [61, 62], human placental tissue [5, 63–65], JEG-3 cells [5], and ovarian tissue in rainbow trout [63]. In the late 1990s, premenopausal women were fed soy isoflavones for approximately 100 days and urine samples were collected to quantify estrogen excretion levels [75]. This last study demonstrated that soy isoflavone consumption may exert cancer-preventive effects by decreasing estrogen synthesis presumably by altering aromatase enzyme activity based upon previously published reports [75]. Finally several synthetic flavones were found to inhibit the aromatization of androstenedione to estrone using human placental microsomes [66] (see Table 1).

### 3.4. 2000–2014: Phytoestrogens Modulation of Aromatase Activity

#### 3.4.1. Increased Interest in Natural Compounds

During the mid to late 1990s there was a trend for many women to turn to “natural” alternative therapies to treat perimenopausal and postmenopausal symptoms [76]. Notably, at this time interval, most women did not elect to take hormone replacement therapy (HRT) and those that did usually stopped or decreased their compliance due to the potential adverse effects of this treatment, especially in reference to cancer issues [77]. Also during this interval recall, in 1991 the women's health initiative (WHI) started and was to run until 2005 but was stopped after an average follow-up of 5.2 years. The WHI investigators' report was published in July of 2002 in the Journal of the American Medical Association (JAMA), which generated confusion due to the fact that the general public did not know how to understand the data [78]. In fact the safety of HRT was challenged, where estrogen plus progestin was shown to increase the risk of invasive breast cancer, coronary heart disease, stroke, and pulmonary embolism, while the risk of colorectal cancer and bone fracture was decreased [78]. This report provided a strong initiative for women to investigate in a more dramatic fashion the use of alternative therapies [76, 79]. Concurrently, Adlercreutz reported in the early 2000s that phytoestrogens had a preventive effect against various cancers and breast cancer specifically. This suggested a dietary link to increase health, in general, and potential prevention of age-related diseases, [80, 81] which supported early reports on this topic [6, 55–59].

For example, during this time, it was estimated that 50% of Americans used some type of dietary supplement on a regular basis and 30 to 50% of women used a “natural” remedy during the postmenopausal interval to address their physical condition in an attempt to improve reproductive-endocrine function while at the same time avoiding the potential adverse effects associated with traditional HRT [76, 79, 82, 83]. Consequently, more women (and men) have turned to natural health remedies such as phytoestrogens (with their polyphenolic chemical structures made in plants) which has become one of the most researched topics in the last decade.

#### 3.4.2. Screening of Natural Compounds (Structure-Function Relationships)

The advancement of technology has enabled investigators to perform what used to be labor-intensive experiments that would take weeks or days and cut the time to conduct these studies by 90% or more. In some cases like molecular and/or gene microarray studies these techniques evolved from the slow paced single compound or isolated gene examination in 1980s and 1990s into high volume and high throughput detection analysis generating pages of data in a very brief time.

For example, before the crystal structure of the aromatase enzyme was reported in 2009, some investigators used three-dimensional modeling techniques of aromatase for structure-based drug design [84]. Others used the concept that dietary phytoestrogens are defined as natural chemicals present in our diet which can mimic or modulate estrogen's hormone action and focused on (a) isoflavones (e.g., soybeans) which constitute the largest group of isoflavonoids (via dietary sources), (b) flavonoids (where over 4,000 compounds have been identified in plant sources), (c) stilbenes, such as resveratrol, and (d) the lignans (i.e., enterolactone and enterodiol) [67]. From such studies numerous reports have confirmed the potential of flavones as aromatase inhibitors [67], such as naringenin, hesperetin, eriodictyol, and naringin. However, in general, flavones are more potent aromatase inhibitors than flavanones [85]. The isoflavonoid molecules, such as biochanin A and formononetin, are strong aromatase inhibitors, while the stilbene and lignan compounds have been shown to be weak aromatase inhibitors [67] (see Table 1).

With regard to modern techniques, Paoletta et al. [86] screened forest phytochemical compounds (constituents of 240 herbs used in traditional Chinese medicine) and identified a number of agents as potential aromatase inhibitors using molecular modeling/docking studies, and the “virtual screening” candidates were subsequently confirmed via aromatase inhibition assays. The flavones, myricetin, and gossypetin displayed inhibitory aromatase activity along with the flavanone, liquiritigenin, which was more potent compared to the aromatase inhibitor, aminoglutethimide [86].

Finally, nutrition mixtures of phytoestrogens and mycotoxins that are commonly found as food contaminants (i.e., zeranol, aflatoxin B, and zearalenone) were tested for their influence on aromatase activity [4, 87]. Taxvig et al. tested 12 food relevant phytoestrogens and found that all of the test materials increased estrogen production and decreased testosterone production, implying that aromatase activity was increased in three different cell lines [4]. Conversely, in another study, zearalenone was found to inhibit aromatase activity using four different cell lines suggesting that not only may molecules from dietary sources impact steroid biosynthesis but also food contaminants may contribute to the modulation of steroid hormone levels [87].

#### 3.4.3. Inhibition or Stimulation of Aromatase Activity

In the 21st century investigators continued to examine phytoestrogen's influence on aromatase but used experimental designs that focused on human breast cancer. In part, this approach was supported by previously published reports demonstrating that isoflavones and other phytoestrogens compounds had antiestrogenic, antiproliferative, and antiaromatase activities. Also, the fact that breast cancer is the predominant type of cancer in industrialized countries, the second leading cause of cancer-related deaths in women, and the increased public awareness of breast cancer campaign galvanized research efforts along this path.

In 2000 Le Bail et al. reported that many phytoestrogens compounds with a B-ring at position-2 were more potent aromatase enzyme inhibitors using human placental microsomes compared to phytochemicals with a B-ring at position-3 [88]. For example, the compounds, flavone, 7-hydroxyflavone, 7-methylflavone, 7,8-dihydroxyflavone, chrysin, apigenin, and naringenin (containing a B-ring at position-2), displayed greater aromatase enzyme inhibition compared to coumestrol, genistein, daidzein, biochanin A, and formononetin containing a B-ring at position-3 [88].

Investigators using MCF-7 cells showed that mammalian lignans (enterolactone and enterodiol) and genistein decreased aromatase enzyme activity suggesting that modulation of local estrogen synthesis is one potential mechanism through which the lignans and genistein protect against breast cancer [68]. Other investigators using human primary mammary fibroblasts and MCF-7 cocultures showed that phytochemicals inhibited aromatase enzyme activity in the following order: biochanin A > genistein > naringenin > apigenin > chrysin [69] (see Table 1). However, in this* in vitro* study, along with other reports, phytoestrogens were shown to increase cell proliferation at concentrations that are not uncommon in blood of individuals using food supplements [4, 89–91]. Some* in vitro* studies reported that genistein induced aromatase enzyme activity along with cell proliferation in a breast cancer model [89, 91]. Although some reports suggested that phytoestrogens tested at higher *μ*M concentrations may decrease cell viability, interact with estrogen receptors, and potentially alter the obtained results in reference to cellular proliferation, that resulted in no effect on aromatase enzyme activity or stimulation of aromatase gene expression [88, 92].

In reference to breast cancer, the disposition of soy isoflavones in normal breast tissue has been reported, where a dietary intervention study of healthy women consuming soy milk for 4 to 5 days before esthetic breast reduction (along with blood sample collection) was performed [93]. Genistein and daidzein serum concentrations ranged from 135 to 2831 nmol/L and from 105 to 1397 nmol/L, respectively, and from 92 to 494 pmol/g and from 22 to 771 pmol/g, respectively, in breast tissue samples. The total isoflavones showed a breast adipose/glandular tissue distribution of 40 : 60 ratio and their concentrations were approximately 30-fold higher compared to 17*β*-estradiol concentrations suggesting that dietary intake of soy products reached levels in breast tissue that may provide health benefits [93].

In brief, reports from human studies examining phytoestrogens provide valuable data from which a comparison can be made as to the confirmation or divergent results seen in* in vitro* and animal studies [49]; since the outcomes or endpoints include no change, a decrease or increase of health benefits related to exposure to phytochemicals. Several reports suggest that dietary soy isoflavone intake was associated with lower risk of occurrence and recurrence among postmenopausal patients with breast cancer positive for estrogen and progesterone receptor [94–96]; the development of phytoestrogen therapy for breast cancer prevention is supported by historical and regional dietary intake patterns [55–59, 80, 81] and the health benefits and the mechanisms by which these polyphenolic molecules exert their positive effects have been reviewed [97–101].

#### 3.4.4. Alteration in Aromatase mRNA Levels

In addition to examining aromatase enzyme activity, some reports have shown that phytoestrogens inhibit aromatase mRNA expression. Rice et al. in 2006 showed that the flavones, apigenin and quercetin, and the isoflavones, genistein, daidzein, and biochanin A, displayed dose-dependent reductions in aromatase mRNA levels with apigenin as the most potent inhibitor of aromatase [70]. Additionally low dose (0.1 *μ*M to 10 *μ*M) combinations of the phytoestrogens were also effective in the reduction of aromatase mRNA levels using human ovarian tissue [70] (see Table 1).

Chi et al. showed that the red clover isoflavone, biochanin A, also inhibits aromatase activity and mRNA expression using breast cancer cell lines (MCF-7 and SK-BR-3) and implicated that the transcriptional control of the CYP19 gene is exon-specific [96]. In addition to biochanin A, the phytoestrogen, genistein, since it did not inhibit aromatase activity but did suppress CYP19A1 gene expression via promoters 1.3 and II, suggested another mechanism by which local estrogen biosynthesis may be regulated [71] (see Table 1). Also examining aromatase promoter mechanisms Wang et al. showed that the food contaminants, zearalenone and zeranol, suppressed aromatase expression through promoters II and 1.3, with zearalenone being the most potent aromatase inhibitor [87]. In investigating other biochemicals, the compound extracted from the tree* Ginkgo biloba* displayed aromatase inhibitory effects at both the enzyme (JEG-3 cells) and CYP19A1 expression levels [102]. This study also examined specific promoter CYP19A1 sequences in recombinant microsomes, which suggested a dose-dependent decline in aromatase gene expression that may have potential applications to breast cancer [102]. Celik et al. tested several vegetables (*Laurus nobilis, Mentha piperita, Crocus sativus*, and* Allium*) as potential aromatase inhibitors via mRNA expression in human non-small cell lung cancer cell lines and found that all were effective CYP19A1 inhibitors presenting a potential role and mechanism of action, where foods may reduce the risk of certain cancers [103]. Conversely, Ye et al. reported that the isoflavone, genistein, induced aromatase activity and increased aromatase mRNA expression in hepatic cells (HepG2) with the concurrent utilization of the CYP19A1 promoters 1.3 and II [104]. Finally, Khan et al. have reviewed studies examining phytochemicals and the potential utility of natural products as regulators of aromatase promoters as therapeutic agents against breast cancer [72] (see Table 1).

## 4. Summary

**Figure 3:**
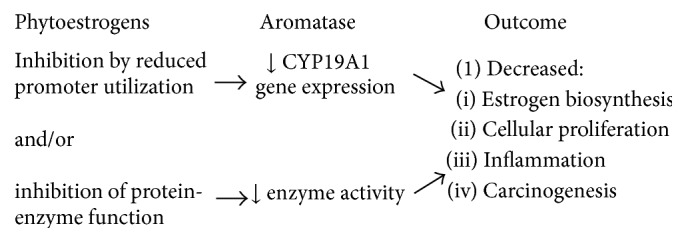
Mechanisms of aromatase inhibition (gene expression and enzyme activity) by phytoestrogens.

The importance of the aromatase enzyme (CYP19A1 gene) to catalyze the conversion of androgens to estrogens in many human tissue sites that are known to stimulate cellular proliferation in breast cancer represents one of the most crucial health issues of our time. Naturally occurring compounds derived from plants which have chemical structures similar to estrogen are classified as phytoestrogens and have received increased research attention because of their potential significance/application in human cancers and other diseases. The inhibition of aromatase (enzyme activity and gene expression) represents one key mechanism of how phytoestrogens may contribute to decreased cancer risk and recurrence (see Figure 3). In general, phytoestrogens act as aromatase inhibitors by (a) decreasing aromatase gene expression (by reducing promoter utilization), (b) inhibiting the aromatase enzyme itself, or (c) in some cases acting at both levels of regulation to diminish local estrogen biosynthesis and cellular proliferation. The findings presented herein are consistent with estrogen's impact on health and phytoestrogens' potential as anticancer agents as well as treatments for other diseases, but well-controlled, large-scale studies are warranted to determine the effectiveness of phytoestrogens on breast cancer and age-related diseases.

## Figures and Tables

**Table 1:** Inhibition of aromatase by phytoestrogen classification.

Aromatase enzyme activity	Gene expression	Phytoestrogen classification	Microsome, cell, cell line, or tissue	Degree of inhibition	Reference
X		Lignans/isoflavonoids	Placental microsomes, JEG-3	Moderate to weak	[5]

X		Flavones	Placental microsomes	Moderate to weak	[60]

X		Flavonoids	Placental microsomes	Moderate to weak	[61]

X		Lignans/flavonoids	Human preadipocytes	Moderate to weak	[62]

X		Flavonoids	Placental microsomes/ovary	Moderate to weak	[63]

X		Flavonoids	Placental microsomes	Moderate to weak	[64]

X		Flavonoids	Placental microsomes	Moderate to weak	[65]

X		Flavonoids	Placental microsomes	Moderate to weak	[66]

X		Lignans/flavonoids/isoflavonoids/stilbenes	Placental microsomes	Moderate to weak	[67]

X		Lignans/flavonoids/isoflavonoids	Placental microsomes, MCF-7, MB 231	Moderate to weak	[68]

X		Lignans/isoflavonoids	MCF-7	Moderate to weak	[69]

X	*▾*	Flavones/isoflavonoids	Granulosa-luteal	Weak	[70]

X	*▾*	Isoflavonoids	MCF-7, SK-BR-3	Moderate to weak	[71]

X	*▾*	Flavonoids/isoflavonoids/stilbenes	Placental microsomes, JEG-3, MCF-7, SK-BR-3	Strong to moderate to weak	[72]

## References

[1] Di Nardo G., Gilardi G. (2013). Human aromatase: perspectives in biochemistry and biotechnology. *Biotechnology and Applied Biochemistry*.

[2] Santen R. J., Brodie H., Simpson E. R., Siiteri P. K., Brodie A. (2009). History of aromatase: saga of an important biological mediator and therapeutic target. *Endocrine Reviews*.

[3] Stocco C. (2012). Tissue physiology and pathology of aromatase. *Steroids*.

[4] Taxvig C., Elleby A., Sonne-Hansen K. (2010). Effects of nutrition relevant mixtures of phytoestrogens on steroidogenesis, aromatase, estrogen, and androgen activity. *Nutrition and Cancer*.

[5] Adlercreutz H., Bannwart C., Wähälä K. (1993). Inhibition of human aromatase by mammalian lignans and isoflavonoid phytoestrogens. *Journal of Steroid Biochemistry and Molecular Biology*.

[6] Adlercreutz H., Mazur W. (1997). Phyto-oestrogens and Western diseases. *Annals of Medicine*.

[7] De Cremoux P., Jacquot Y., Preedy V. R. (2013). Isoflavones and human estrogen receptor: when plants synthesis mammalian hormone minetics. *Isoflavones: Chemistry, Analysis, Function and Effects*.

[8] Karkola S., Wähälä K. (2009). The binding of lignans, flavonoids and coumestrol to CYP450 aromatase: a molecular modelling study. *Molecular and Cellular Endocrinology*.

[9] Ingram D., Sanders K., Kolybaba M., Lopez D. (1997). Case-control study of phyto-oestrogens and breast cancer. *The Lancet*.

[10] Smith C. L., Knobil E., Neill J. D. (1999). Estrogens, overview. *Encyclopedia of Reproduction*.

[11] Meyer A. S. (1955). Conversion of 19-hydroxy-Δ^4^-androstene-3,17-dione to estrone by endocrine tissue. *Biochimica et Biophysica Acta*.

[12] Meyer A. S., Hayano M., Lindberg M. C., Gutt M., Rogers O. G. (1955). The conversion of *δ*4-androstene-3,17-dione-4-C14 and dehydroepiandrosterone by bovine adrenal homogenate preparations. *Acta Endocrinologica*.

[13] Ryan K. J. (1959). Biological aromatization of steroids. *The Journal of Biological Chemistry*.

[14] Thompson E. A., Siiteri P. K. (1974). The involvement of human placental microsomal cytochrome P-450 in aromatization. *The Journal of Biological Chemistry*.

[15] Grodin J. M., Siiteri P. K., MacDonald P. C. (1973). Source of estrogen production in postmenopausal women. *The Journal of Clinical Endocrinology & Metabolism*.

[16] Hemsell D. L., Edman C. D., Marks J. F., Siiteri P. K., MacDonald P. C. (1977). Massive extraglandular aromatization of plasma androstenedione resulting in feminization of a prepubertal boy. *The Journal of Clinical Investigation*.

[17] Boon W. C., Chow J. D. Y., Simpson E. R. (2010). The multiple roles of estrogens and the enzyme aromatase. *Progress in Brain Research*.

[18] Ghosh D., Griswold J., Erman M., Pangborn W. (2009). Structural basis for androgen specificity and oestrogen synthesis in human aromatase. *Nature*.

[19] Kamat A., Hinshelwood M. M., Murry B. A., Mendelson C. R. (2002). Mechanisms in tissue-specific regulation of estrogen biosynthesis in humans. *Trends in Endocrinology and Metabolism*.

[20] Lephart E. D. (1996). A review of brain aromatase cytochrome P450. *Brain Research Reviews*.

[21] Lobo R. A., Davis S. R., De Villiers T. J. (2014). Prevention of diseases after menopause. *Climacteric*.

[22] Marsh D. A., Brodie H. J., Garrett W., Tsai-Morris C.-H., Brodie A. M. H. (1985). Aromatase inhibitors. Synthesis and biological activity of androstenedione derivatives. *Journal of Medicinal Chemistry*.

[23] Olin J. L., St. Pierre M. (2014). Aromatase inhibitors in breast cancer prevention. *Annals of Pharmacotherapy*.

[24] Simpson E. R., Clyne C., Rubin G. (2002). Aromatase—a brief overview. *Annual Review of Physiology*.

[25] Manach C., Williamson G., Morand C., Scalbert A., Rémésy C. (2005). Bioavailability and bioefficacy of polyphenols in humans. I. Review of 97 bioavailability studies. *The American Journal of Clinical Nutrition*.

[26] Manach C., Scalbert A., Morand C., Rémésy C., Jiménez L. (2004). Polyphenols: food sources and bioavailability. *The American Journal of Clinical Nutrition*.

[27] Cheruiyot E. K., Mumera L. M., Ng'etich W. K., Hassanali A., Wachira F. (2007). Polyphenols as potential indicators for drought tolerance in tea (*Camellia sinensis* L.). *Bioscience, Biotechnology and Biochemistry*.

[28] Salah N., Miller N. J., Paganga G., Tijburg L., Bolwell G. P., Rice-Evans C. (1995). Polyphenolic flavanols as scavengers of aqueous phase radicals and as chain-breaking antioxidants. *Archives of Biochemistry and Biophysics*.

[29] Spencer J. P. E., Abd El Mohsen M. M., Minihane A.-M., Mathers J. C. (2008). Biomarkers of the intake of dietary polyphenols: strengths, limitations and application in nutrition research. *British Journal of Nutrition*.

[30] Liu C.-M., Chen C.-Y., Lin Y.-W. (2014). Estimation of tea catechin levels using micellar electrokinetic chromatography: a quantitative approach. *Food Chemistry*.

[31] Butt M. S., Imran A., Sharif M. K. (2014). Black tea polyphenols: a mechanistic treatise. *Critical Reviews in Food Science and Nutrition*.

[32] Meydani M., Hasan S. T. (2010). Dietary polyphenols and obesity. *Nutrients*.

[33] Khurana S., Venkataraman K., Hollingsworth A., Piche M., Tai T. C. (2013). Polyphenols: benefits to the cardiovascular system in health and in aging. *Nutrients*.

[34] Wang S., Moustaid-Moussa N., Chen L. (2014). Novel insights of dietary polyphenols and obesity. *The Journal of Nutritional Biochemistry*.

[35] Evans J. A., Johnson E. J. (2010). The role of phytonutrients in skin health. *Nutrients*.

[36] Takaoka M. J. (1940). Of the phenolic substances of white hellebore (*Veratrum gandiflorum* Loes. fil.). *Journal of the Faculty of Science, Hokkaido University*.

[37] Nonomura S., Kanagawa H., Makimoto A. (1963). Chemical constituents of polygonaceous plants. I. Studies on the components of Koj O-Kon. (Polygonum Cuspidatum Sieb. Et Zucc.). *Yakugaku Zasshi*.

[38] Siemann E. H., Creasy L. L. (1992). Concentration of the phytoalexin resveratrol in wine. *American Journal of Enology and Viticulture*.

[39] Bertelli A. A. E., Giovannini L., Giannessi D. (1995). Antiplatelet activity of synthetic and natural resveratrol in red wine. *International Journal of Tissue Reactions*.

[40] Jang M., Cai L., Udeani G. O. (1997). Cancer chemopreventive activity of resveratrol, a natural product derived from grapes. *Science*.

[41] Tomé-Carneiro J., Larrosa M., González-Sarrías A., Tomás-Barberán F. A., García-Conesa M. T., Espín J. C. (2013). Resveratrol and clinical trials: the crossroad from in vitro studies to human evidence. *Current Pharmaceutical Design*.

[42] Pezzuto J. M. (2011). The phenomenon of resveratrol: redefining the virtues of promiscuity. *Annals of the New York Academy of Sciences*.

[43] Lam Y. Y., Peterson C. M., Ravussin E. (2013). Resveratrol and calorie restriction: data from rodents to humans. *Experimental Gerontology*.

[44] Turan B., Tuncay E., Vassort G. (2012). Resveratrol and diabetic cardiac function: focus on recent in vitro and in vivo studies. *Journal of Bioenergetics and Biomembranes*.

[45] Bhatt J. K., Thomas S., Nanjan M. J. (2012). Resveratrol supplementation improves glycemic control in type 2 diabetes mellitus. *Nutrition Research*.

[46] Crandall J. P., Oram V., Trandafirescu G. (2012). Pilot study of resveratrol in older adults with impaired glucose tolerance. *The Journals of Gerontology A: Biological Sciences & Medical Sciences*.

[47] Park E.-J., Pezzuto J. M. (2015). The pharmacology of resveratrol in animals and humans. *Biochimica et Biophysica Acta*.

[48] Setchell K. D. R., Clerici C. (2010). Equol: history, chemistry, and formation. *The Journal of Nutrition*.

[49] Lephart E. D., Preedy V. R. (2013). Isoflavones and prenatal exposure to equol. *Isoflavones: Chemistry, Analysis, Function and Effects*.

[50] Lund T. D., Blake C., Bu L., Hamaker A. N., Lephart E. D. (2011). Equol an isoflavonoid: potential for improved prostate health, *in vitro* and *in vivo* evidence. *Reproductive Biology and Endocrinology*.

[51] Gopaul R., Knaggs H. E., Lephart E. D. (2012). Biochemical investigation and gene analysis of equol: a plant and soy-derived isoflavonoid with antiaging and antioxidant properties with potential human skin applications. *Biofactors*.

[52] Lephart E. D. (2013). Protective effects of equol and their polyphenolic isomers against dermal aging: microarray/protein evidence with clinical implications and unique delivery into human skin. *Pharmaceutical Biology*.

[53] Goldin B. R., Adlercreutz H., Dwyer J. T., Swenson L., Warram J. H., Gorbach S. L. (1981). Effect of diet on excretion of estrogens in premenopausal and post-menopausal women. *Cancer Research*.

[54] Goldin B. R., Adlercreutz H., Gorbach S. L. (1982). Estrogen excretion patterns and plasma levels in vegetarian and omnivorous women. *The New England Journal of Medicine*.

[55] Aldercreutz H. (1984). Does fiber-rich food containing animal lignan precursors protect against both colon and breast cancer? An extension of the ‘fiber hypothesis’. *Gastroenterology*.

[56] Goldin B. R., Adlercreutz H., Gorbach et A. S. L. (1986). The relationship between estrogen levels and diets of Caucasian American and Oriental immigrant women. *The American Journal of Clinical Nutrition*.

[57] Axelson M., Sjovall J., Gustaffson B. E., Setchell K. D. R. (1984). Soya—a dietary source of the non-steroidal oestrogen equol in man and animals. *Journal of Endocrinology*.

[58] Setchell K. D. R., Borriello S. P., Hulme P., Kirk D. N., Axelson M. (1984). Nonsteroidal estrogens of dietary origin: possible roles in hormone-dependent disease. *The American Journal of Clinical Nutrition*.

[59] Adlercreutz H. (1990). Diet, breast cancer, and sex hormone metabolism. *Annals of the New York Academy of Sciences*.

[60] Kellis J. T., Vickery L. E. (1984). Inhibition of human estrogen synthetase (aromatase) by flavones. *Science*.

[61] Campbell D. R., Kurzer M. S. (1993). Flavonoid inhibition of aromatase enzyme activity in human preadipocytes. *Journal of Steroid Biochemistry and Molecular Biology*.

[62] Wang C. F., Mäkelä T., Hase T., Adlercreutz H., Kurzer M. S. (1994). Lignans and flavonoids inhibit aromatase enzyme in human preadipocytes. *Journal of Steroid Biochemistry and Molecular Biology*.

[63] Pelissero C., Lenczowski M. J. P., Chinzi D., Davail-Cuisset B., Sumpter J. P., Fostier A. (1996). Effects of flavonoids on aromatase activity, an in vitro study. *Journal of Steroid Biochemistry and Molecular Biology*.

[64] Le Bail J. C., Laroche T., Marre-Fournier F., Habrioux G. (1998). Aromatase and 17*β*-hydroxysteroid dehydrogenase inhibition by flavonoids. *Cancer Letters*.

[65] Jeong H.-J., Shin Y. G., Kim I.-H., Pezzuto J. M. (1999). Inhibition of aromatase activity by flavonoids. *Archives of Pharmacal Research*.

[66] Ibrahim A.-R., Abul-Hajj Y. J. (1990). Aromatase inhibition by flavonoids. *Journal of Steroid Biochemistry and Molecular Biology*.

[67] Basly J.-P., Lavier M.-C. C. (2005). Dietary phytoestrogens: potential selective estrogen enzyme modulators?. *Planta Medica*.

[68] Brooks J. D., Thompson L. U. (2005). Mammalian lignans and genistein decrease the activities of aromatase and 17*β*-hydroxysteroid dehydrogenase in MCF-7 cells. *Journal of Steroid Biochemistry and Molecular Biology*.

[69] van Meeuwen J. A., Korthagen N., de Jong P. C., Piersma A. H., van den Berg M. (2007). Antiestrogenic effects of phytochemicals on human primary mammary fibroblasts, MCF-7 cells and their co-cultures. *Toxicology and Applied Pharmacology*.

[70] Rice S., Mason H. D., Whitehead S. A. (2006). Phytoestrogens and their low dose combinations inhibit mRNA expression and activity of aromatase in human granulosa-luteal cells. *Journal of Steroid Biochemistry and Molecular Biology*.

[71] Wang Y., Gho W. M., Chan F. I., Chen S., Leung L. K. (2008). The red clover (*Trifolium pratense*) isoflavone biochanin A inhibits aromatase activity and expression. *British Journal of Nutrition*.

[72] Khan S. I., Zhao J., Khan I. A., Walker L. A., Dasmahapatra A. K. (2011). Potential utility of natural products as regulators of breast cancer-associated aromatase promoters. *Reproductive Biology and Endocrinology*.

[73] Santen R. J., Demers L. M., Adlercreutz H. (1989). Inhibition of aromatase with CGS-16949A in postmenopausal women. *Journal of Clinical Endocrinology and Metabolism*.

[74] Masamura S., Adlercreutz H., Harvey H. (1995). Aromatase inhibitor development for treatment of breast cancer. *Breast Cancer Research and Treatment*.

[75] Xu X., Duncan A. M., Merz B. E., Kurzer M. S. (1998). Effects of soy isoflavones on estrogen and phytoestrogen metabolism in premenopausal women. *Cancer Epidemiology Biomarkers and Prevention*.

[76] Messina M. J. (2002). Soy foods and soybean isoflavones and menopausal health. *Nutrition in Clinical Care*.

[77] White J. P., Schilling J. S. (2000). Postmenopausal hormone replacement: historical perspectives and current concerns. *Clinical Excellence for Nurse Practitioners*.

[78] Rossouw J. E., Anderson G. L., Prentice R. L. (2002). Risks and benefits of estrogen plus progestin in healthy postmenopausal women: principal results from the women's health initiative randomized controlled trial. *The Journal of the American Medical Association*.

[79] Fitzpatrick L. A. (2003). Soy isoflavones: hope or hype?. *Maturitas*.

[80] Adlercreutz H. (2002). Phyto-oestrogens and cancer. *The Lancet Oncology*.

[81] Adlercreutz H. (2002). Phytoestrogens and breast cancer. *The Journal of Steroid Biochemistry and Molecular Biology*.

[82] Mackey R., Evans R. M. (1995). Phytoestrogens and the menopause. *Climacteric*.

[83] Messerer M., Johansson S.-E., Wolk A. (2001). Sociodemographic and health behaviour factors among dietary supplement and natural remedy users. *European Journal of Clinical Nutrition*.

[84] Karkola S., Höltje H.-D., Wähälä K. (2007). A three-dimensional model of CYP19 aromatase for structure-based drug design. *Journal of Steroid Biochemistry and Molecular Biology*.

[85] Sanderson J. T., Hordijk J., Denison M. S., Springsteel M. F., Nantz M. H., van den Berg M. (2004). Induction and inhibition of aromatase (CYP19) activity by natural and synthetic flavonoid compounds in H295R human adrenocortical carcinoma cells. *Toxicological Sciences*.

[86] Paoletta S., Steventon G. B., Wildeboer D., Ehrman T. M., Hylands P. J., Barlow D. J. (2008). Screening of herbal constituents for aromatase inhibitory activity. *Bioorganic & Medicinal Chemistry*.

[87] Wang Y. E., Wong T. Y., Chan F. L., Chen S., Leung L. K. (2014). Assessing the effect of food mycotoxins on aromatase by using a cell-based system. *Toxicology in Vitro*.

[88] Le Bail J.-C., Champavier Y., Chulia A.-J., Habrioux G. (2000). Effects of phytoestrogens on aromatase, 3*β* and 17*β*-hydroxysteroid dehydrogenase activities and human breast cancer cells. *Life Sciences*.

[89] van Duursen M. B. M., Nijmeijer S. M., de Morree E. S., de Jong P. C., van den Berg M. (2011). Genistein induces breast cancer-associated aromatase and stimulates estrogen-dependent tumor cell growth in in vitro breast cancer model. *Toxicology*.

[90] van Duursen M. B. M., Smeets E. E. J. W., Rijk J. C. W., Nijmeijer S. M., van den Berg M. (2013). Phytoestrogens in menopausal supplements induce ER-dependent cell proliferation and overcome breast cancer treatment in an in vitro breast cancer model. *Toxicology and Applied Pharmacology*.

[91] Richter D. U., Mylonas I., Toth B. (2009). Effects of phytoestrogens genistein and daidzein on progesterone and estrogen (estradiol) production of human term trophoblast cells in vitro. *Gynecological Endocrinology*.

[92] Tiemann U., Schneider F., Vanselow J., Tomek W. (2007). In vitro exposure of porcine granulosa cells to the phytoestrogens genistein and daidzein: effects on the biosynthesis of reproductive steroid hormones. *Reproductive Toxicology*.

[93] Bolca S., Urpi-Sarda M., Blondeel P. (2010). Disposition of soy isoflavones in normal human breast tissue. *The American Journal of Clinical Nutrition*.

[94] Kang X. M., Zhang Q. Y., Wang S. H., Huang X., Jin S. (2010). Effect of soy isoflavones on breast cancer recurrence and death for patients receiving adjuvant endocrine therapy. *Canadian Medical Association Journal*.

[95] Rice S., Whitehead S. A. (2008). Phytoestrogens oestrogen synthesis and breast cancer. *The Journal of Steroid Biochemistry and Molecular Biology*.

[96] Chi F., Wu R., Zeng Y.-C., Xing R., Liu Y., Xu Z.-G. (2013). Post-diagnosis soy food intake and breast cancer survival: a meta-analysis of cohort studies. *Asian Pacific Journal of Cancer Prevention*.

[97] Liu M. M., Huang Y., Wang J. (2012). Developing phytoestrogens for breast cancer prevention. *Anti-Cancer Agents in Medicinal Chemistry*.

[98] Sirotkin A. V., Harrath A. H. (2014). Phytoestrogens and their effects. *European Journal of Pharmacology*.

[99] Minatoya M., Kutomi G., Asakura S. (2013). Equol, adiponectin, insulin levels and risk of breast cancer. *Asian Pacific Journal of Cancer Prevention*.

[100] Lephart E. D. (2014). Review: anti-oxidant and anti-aging properties of equol in prostate health (BPH). *Open Journal of Endocrine and Metabolic Diseases*.

[101] Ajdžanović V. Z., Medigović I. M., Pantelić J. B., Milošević V. L. (2014). Soy isoflavones and cellular mechanics. *Journal of Bioenergetics and Biomembranes*.

[102] Kim M. J., Park Y. J., Chung K. H., Oh S. M. (2013). The inhibitory effects of the standardized extracts of *Ginkgo biloba* on aromatase activity in JEG-3 human choriocarcinoma cells. *Phytotherapy Research*.

[103] Celik G., Akca H., Sen A. (2013). Investigation of aromotase inhibition by several dietary vegetables in human non-small cell lung cancer cell lines. *Turkish Journal of Biochemistry*.

[104] Ye L., Chan M. Y., Leung L. K. (2009). The soy isoflavone genistein induces estrogen synthesis in an extragonadal pathway. *Molecular and Cellular Endocrinology*.

